# Everybody's Page

**Published:** 1889-11-30

**Authors:** 


### Everybody's Page.

An old woman has died at Anglesea who was married 74 years ago, and she had quite recently cut three teeth.

The experiments of Prof. Cohn, of Breslau, have proved that the heating of masses of damp hay to such a temperature as to cause spontaneous combustion is caused by fungus.

The Calcutta Public Health Society evidently mean to do all they can to further leper legislation. They want to stop all chance of lepers being employed in washing clothes or preparing any sort of food, but the native population object to the legislation affecting any but paupers.

Cooking pots are often in fault when food is nasty, and often cause a whole family to suffer from indigestion. Copper utensils should be lined with tin, and should be tinned every three months. After tinning, bran should be boiled in them before they are used for food. Sir W. Moore says these precautions are specially necessary in India.

Coffee jelly is one of the newest and nicest delicacies for the sick-room. Put 2oz. of gelatine to soak in a pint of water; when it has dissolved add ½lb. of castor sugar, half a pint of brandy, juice of one lemon, and about a pint and a half of strong coffee. When the sugar has melted add the whites and crushed shells of three eggs. Whisk it quickly on the fire, pass it through the jelly bag, pour it into a damp mould, and leave it to set.

The embalming of dead bodies is a very ancient practice, and is described by Herodotus. Three different persons appear to have been employed in making an embalmment. A designer, whose office it was to trace the lines of dissection on the body; a dissector, by whom the operation of preparing the body was performed; and the embalmer proper. The lines of dissection were indicated by law, and the operation was performed with a sharply-pointed instrument of Ethiopian stone.

Dr. E. Golay, the French physician, in his recent work has been advocating a wider basis to the education of the fair sex. There is, he considers, one thing especially wanting in the tuition of the present day, and he is anxious that young girls when at school should be instructed in the art of bringing up children, which to his mind is the most useful of all the subjects which they should learn. He points out very truly that the health of a child is to a great extent what the mother makes it. No doubt the health of many a child suffers, and has suffered, and for that matter always will suffer, from its mother's ignorance. Whether Dr. Golay's wishes will ever be gratified the future alone can show.

According to the same authority, yellow fever, cholera, smallpox, splenic fever, scarlet fever, diphtheria, and other contagious diseases can be communicated, many years after the burial of the dead, by the germs in the grave, or in the earth surrounding it, which retain their vitality and power to spread disease. An experiment with some soil from a cemetery in Brazil, where victims of yellow fever had been buried, gave a curious corroboration of this fact. Some of the earth was taken, and, after being dried, was placed in a cage with a guinea-pig, which sickened and died within five days. The post-mortem showed that the death was undoubtedly due to yellow fever. The brain and the intestines were stained yellow, and all the tissues presented the characteristic changes which are caused by yellow fever. What an opportunity for experiments was lost after the plague of London, and how simply the surplus feline population might have been reduced.

Only one-fourth of the population of England reach threescore and ten, only about fifteen in 100,000 become centenarians.

Thirst, which is often a symptom of an unsuspected disease, is best allayed by citrate of magnesia or nitrate of potash.

The Tahkalis, a North American Indian tribe, like decayed substances as food. They only eat the flesh of quadrupeds or fish when it is in a state of complete putrefaction, and we are told of a wild tribe who, in boiling their wild rice to eat, mix with it the excrements of rabbits, and esteem this ingredient a delicacy.

A form of food used in action by the German army is called "iron ration." It is a peculiar kind of bread, in the shape of small cubes the size of a chocolate-drop, made of fine wheat bread strongly spiced, and calculated to keep for a long time. When taken into the mouth it quickly softens, and is both palatable and nutritious. It is chiefly intended for forced marches, when there is no time for camping and cooking.

People are frequently to be heard asking at a bar for a pint of "half-and-half." Some generations back it was as common to hear people ask for a pint of "three-thirds." This was a third of beer, ale, and twopenny, each drawn into one tankard from a different cask. The trouble of going to three different casks in order to draw the "three-thirds" originated the idea of making porter, which combined the flavour of the three.

One of the minerals of America which is coming rapidly into greater use is soapstone. It is employed variously; for instance, in the dressing of leather, etc.; in some places headstones are made of it; the Chinese carve their idols out of it. Its greatest use is as an adulterant, for, being very light, it mixes well in the manufacture of soap, rubber, and paper. One of the most familiar forms we see it in in this country is that of little tablets, which tailors use for marking alterations on our garments.

In his address on "The Sanitary Disposition of the Dead," Dr. C. A. Harvey, of New York, gave some very interesting information. He stated that many of the old burying grounds of Europe have received so great a number of human remains as to raise the top soil from one to four feet above the surrounding ground. Many of such burial places have been the fruitful source of epidemics and devastating plagues which have well nigh depopulated the regions about them. Notable among these was the Cemetery of the Innocents, in Paris, the ground of which had been so filled with buried dead as to raise it some three feet above its normal level. The result was the breaking out of a terribly devastating plague.

A series of experiments made by Von Gregor Kraus, the results of which are embodied in a monograph recently published at Leipzig, have led him to believe that the use of tannin to leaves is to protect them, either from rotting or from being eaten. He found that leaves which are not green are not capable of producing tannin, and that fallen leaves contain as much tannin as they do during the best time of their growth. The outer leaves of a plant, exposed to direct sunlight, contain more tannin than the inner leaves which, of course, are more or less shielded from the rays of the sun; yet, curiously, during the germination, in the dark, of seeds containing tannin there is no diminution, but an increase in the amount of tannin.

